# Individualized Analysis and Treatment of Difficult Weaning From Ventilation Following Open Cardiac Surgery in Young Children With Congenital Heart Disease

**DOI:** 10.3389/fcvm.2022.768904

**Published:** 2022-06-01

**Authors:** Xiaoming Wu, Jinlan Chen, Chukwuemeka Daniel Iroegbu, Jian Liu, Ming Wu, Xia Xie, Kun Xiang, Xun Wu, Wangping Chen, Peng Huang, Wenwu Zhou, Chengming Fan, Jinfu Yang

**Affiliations:** ^1^Department of Cardiovascular Surgery, The Second Xiangya Hospital, Central South University, Changsha, China; ^2^Department of Cardiothoracic Surgery, Hunan Children's Hospital, Changsha, China; ^3^Department of the Cardiovascular Surgery of the Hunan Provincial People's Hospital, Changsha, China

**Keywords:** individualized treatment, difficult weaning, open cardiac surgery, pediatric patients, congenital heart disease, slide

## Abstract

**Aims:**

The study explores the leading causes of postoperative extubation difficulties in pediatric patients (neonates and toddlers) with congenital heart diseases and establishes individualized treatment for different reasons.

**Method:**

We retrospectively analyzed medical records of 4,971 pediatric patients with congenital heart defects treated in three tertiary Congenital Heart Disease Centres in China from January 2005 to December 2020, from whom we selected those with difficulty extubation but successful weaning during the postoperative period. Next, we performed an analysis of risk factors and reported the combined experience of individualized treatment for successful extubation.

**Results:**

Seventy-five pediatric patients were identified in our database, among whom 23 had airway stenosis, 17 had diaphragmatic dysfunction, and 35 had pulmonary infection. The patients were all successfully weaned from the ventilator after an individualized treatment plan. In addition, the intubation time in the airway stenosis group was 17.7 ± 9.0, 33.6 ± 13.9 days in the diaphragmatic dysfunction group, and 11.9 ± 3.8 days in the pulmonary infection group.

**Conclusion:**

Given the primary reasons for difficult weaning following open-heart surgery in pediatric patients with congenital heart diseases, an individualized treatment scheme can achieve the ideal therapeutic effect where patients can be weaned faster with a shorter intubation period.

## Introduction

Mechanical ventilation, an effective treatment strategy used for most congenital heart disease following pediatric or adult cardiac surgery, is essential for post-operative recovery. In most cases, pediatric patients are post-operatively successfully weaned as planned; however, about 6–27% scenarios with weaning difficulties have been reported (1). Weaning difficulty is defined as the time required for the patient to pass the spontaneous breathing test SBT, or to successfully separate from the ventilator after the first SBT, which usually takes at least 24 h or longer (2, 3). Thus, in such cases, complications such as ventilator-associated lung injury, ventilator-dependency, malnutrition, and ventilator-associated pneumonia are frequently encountered (4–7), leading to higher extubation failure rates, longer hospital stays, and higher medical costs and consumption of medical resources (8).

Weaning difficulties after surgery for infants and children with congenital heart diseases are related to several factors, such as unstable hemodynamics following cardiac surgery, pulmonary hypertension, and delayed awakening (9, 10). In most cases, these patients often have accompanying structural health alignments such as tracheal or bronchial stenosis (11), lung infections, and acquired complications such as post-operative diaphragmatic dysfunctions (12), which have been identified as influencing factors of weaning difficulty in previous studies. Although there are some studies in the literature analyzing risk factors for extubation failure and how this affects patient outcomes, but there are few studies analyzing the treatment of failed weaning. In the study herein, we mainly explored the main causes of failure to wean from invasive mechanical ventilation following cardiac surgery in neonatal and pediatric patients. All hemodynamic causes were excluded, focusing on respiratory (congenital or acquired) causes. The three main causes identified are: airway stenosis (congenital or secondary), diaphragm dysfunction (partial or complete), and pneumonias. Although these factors can also be seen in ICU pediatric patients with non-cardiac surgery; however, the clinical characteristics after cardiac surgery are different, often accompanied by cardiac function inhibition, mediastinal and thoracic tissue destruction, organ damage, and systemic inflammation after cardiopulmonary bypass. Thus, the treatment is more complex, and the prognosis is less sanguine. It is extremely challenging as the combination conditions may require a change or alteration in conventional strategy. During the past 10 years, apart from simply extending mechanical ventilation or tracheotomy, some cardiac surgery centers pioneered many of the techniques that are currently used (13–15). However, a generally accepted individualized treatment plan is yet to be introduced or adopted.

Hence, in the study herein, we reviewed the surgical treatment of pediatric patients with congenital heart diseases from January 2005 to December 2020. Also, primary factors affecting weaning (except cardiac reasons including residual shunts) were statistically analyzed with proposed individualized treatment plans per cause or reasons.

## Methods

### Study Population

This retrospective study was conducted in pediatric patients with congenital heart defects using the hospital information system. Records of congenital heart surgery cases performed at the Department of Cardiac Surgery of the Second Xiangya Hospital, Hunan Children's Hospital, and Hunan People's Hospital between January 2005 and December 2020 were reviewed. We selected cases from the database that met the following criteria: 1. Age <36 months, 2. diagnosed with congenital heart defects, 3. successfully wean after receiving invasive mechanical ventilation for more than 7 days, 4. no specialist conditions affecting weaning, such as postoperative cardiac hemodynamic instability, pulmonary hypertension, and delayed awakening. Exclusion criteria: 1. prolonged weaning due to incomplete correction of cardiac malformation, 2. postoperative hemodynamic instability or vasoactive-inotropic score (VIS) >15, 3. Postoperative delayed chest closure, 4. Postoperative ECMO assistance is required, 5. Short-term secondary surgery was performed. All patients were approved by the Ethics Committee of the Second Xiangya Hospital, Hunan Children's Hospital, and Hunan People's Hospital.

### Variables

Demographic and clinical characteristics data were extracted from medical records for further details, including age, gender, weight, diagnoses according to International Classifications of Disease 10th revision, history and physical examination findings, ventilation settings, extubation attempts, various therapies, duration of mechanical ventilation in calendar days, etc.

Departmental criteria for weaning were: 1. good mental status, strong autonomic respiratory capacity; 2. hemodynamic stability, VIS <15, 3. ventilator parameters: CPAP/PS mode, oxygen concentration ≤0.5, PEEP ≤5 cmH2O, oxygenation index ≥150 mmHg, PS ≤14 cmH2O, tidal volume ≥7 ml/Kg; 4. No electrolyte disturbance and acid-base imbalance, arterial blood lactate ≤3 mmol/L, 5. No severe tissue edema.

Definition of weaning difficulty: the application of low-level CPAP/PS mode for the deextubation test, the child cannot meet the above withdrawal criteria, or the withdrawal of the ventilator 24 h autonomic breathing cannot maintain effective ventilation and/or oxygenation, the need for re-intubation for mechanical ventilation.

### Statistics

All the data are presented as mean ± standard error of the mean (mean ± SE). For all the experimental data, statistical calculations were performed using the Statistical Product and Service Solutions 14.0 software (SPSS Institute). *X*^2^ test was performed to determine differences between the groups. A *p*-value < 0.05 was considered statistically significant.

## Results

### Factors Associated With Weaning Difficulty

During the study period, a total of 4,971 pediatric patients underwent open heart surgery were retrieved from the hospital information database. After screening, there were 75 patients had weaning difficulties identified in our study.

- There were 23 stenotic tracheobronchial cases, (12 females and 11 males), with an age of 7.09 ± 2.19 months and a weight of 7.67 ± 1.20 Kg. Preoperative diagnosis included 17 pulmonary sling cases, 3 aortic ring cases, and 1 case of CAVSD, VSD, and PDA.
- There were 17 cases of diaphragmatic dysmotility (11 cases of bilateral and 6 cases of unilateral diaphragmatic dysmotility), including 6 females and 11 males, with an age of 9.76 ± 4.78 months and weight of 7.80 ± 1.94 Kg. Preoperative diagnosis included 6 TAPVC cases, 4 TOF cases, 1 TGA case, and 2 cases of DORV, PA, and VSD.
- There were 35 severe pneumonia cases, with an average age and weight of 3.51 ± 1.33 months and 5.58 ± 1.18 Kg. Preoperative diagnosis was VSD with pulmonary hypertension in 21 cases, ASD with pulmonary hypertension in 2 cases, TAPVC in 5 cases, CAVSD in 4 cases, TGA in 2 cases, and 1 PA case (Table 1).

**Table 1:** The profiles of the patients preoperatively.

	**Stenotic tracheobronchial**	**Diaphragmatic dysmotility**	**Severe pneumonia**	***P*-value**
Case (*n*)	23	17	35	0.0847
Gender (male/female)	11/12	11/6	15/20	0.3303
Age (month)	7.09 ± 2.19	9.76 ± 4.78	3.51 ± 1.33	0.4661
Body weight (Kg)	7.67 ± 1.20	7.80 ± 1.94	5.58 ± 1.18	0.8968
Pulmonary sling (*n*)	17			<0.0001
Aortic ring (*n*)	3			0.0442
CAVSD (*n*)	1		4	0.3043
VSD (*n*)	1	2	21	0.0019
ASD (*n*)			2	0.3292
PDA (*n*)	1			0.3336
TAPVC (*n*)		6	5	0.0299
TOF (*n*)		4		0.003
TGA (*n*)		1	2	0.5206
PA (*n*)		2	1	0.1898
DORV (*n*)		2		0.0435

*CAVSD complete atrioventricular septal defect, VSD ventricular septal defect, ASD atrial septal defect, PDA patent ductus arteriosus, TAPVC total anomalous pulmonary venous connection, TOF tetralogy of Fallot, TGA transposition of the great arteries, PA pulmonary atresia, DORV double outlet of right ventricular*.

### Confirmation of the Diagnosis

**Figure 1:**
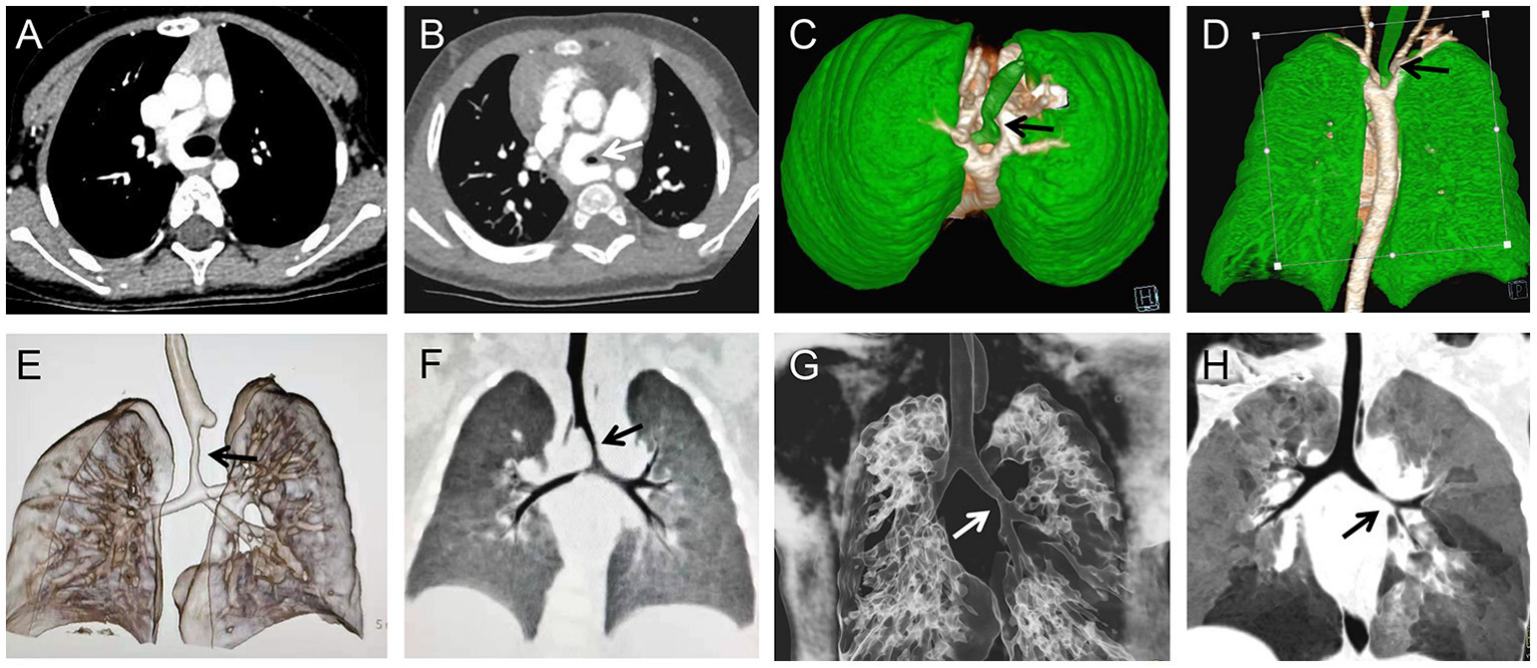
Pulmonary CT and three-dimensional reconstruction of the airway, preoperatively showing the airway stenosis (arrows) was caused by pulmonary artery sling **(A,B)**, double aortic arch **(C,D)**, and a representative image of the stenosis (arrows) located in the trachea **(E,F)** and bronchus **(G,H)**.

In the airway stenosis group, 20 were identified concomitantly with the diagnosis of their heart defect (17 ulmonary artery sling, 3 aortic ring) due to routine preoperative examination and 3 were identified subsequent to repair of their heart defects; compression stenosis of the left main bronchus was found by fiberoptic bronchoscopy in 4 cases. In the diaphragmatic dysfunction group, all cases were confirmed postoperatively by aside chest x-ray. In the pulmonary infection group, all cases had a history of multiple pediatric hospitalizations and underwent routine chest x-ray examination, and most of them were found to have pulmonary exudation; Twenty-two cases underwent lung CT examination before the surgery (Figure 1; Table 2).

**Table 2:** Confirmation of the diagnosis and mechanical ventilation duration.

	**Check**	**Pre-operative confirmation**	**Postoperative confirmation**	**Band time (days)**
Airway stenosis	CT	20	3	17.7
	Bronchoscopy	0	4	
Diaphragmatic dysfunction	CXR	0	17	33.6
Pulmonary infection	CT	22	0	11.9
	CXR	35	0	

### Individualized Treatment

In addition to routine ICU treatment, we also adopted different treatment measures per different causes. In some cases, we integrated multiple treatment measures to establish an individualize treatment plan. More details are detailed below:

*Airway Stenosis Group*

**Figure 2:**
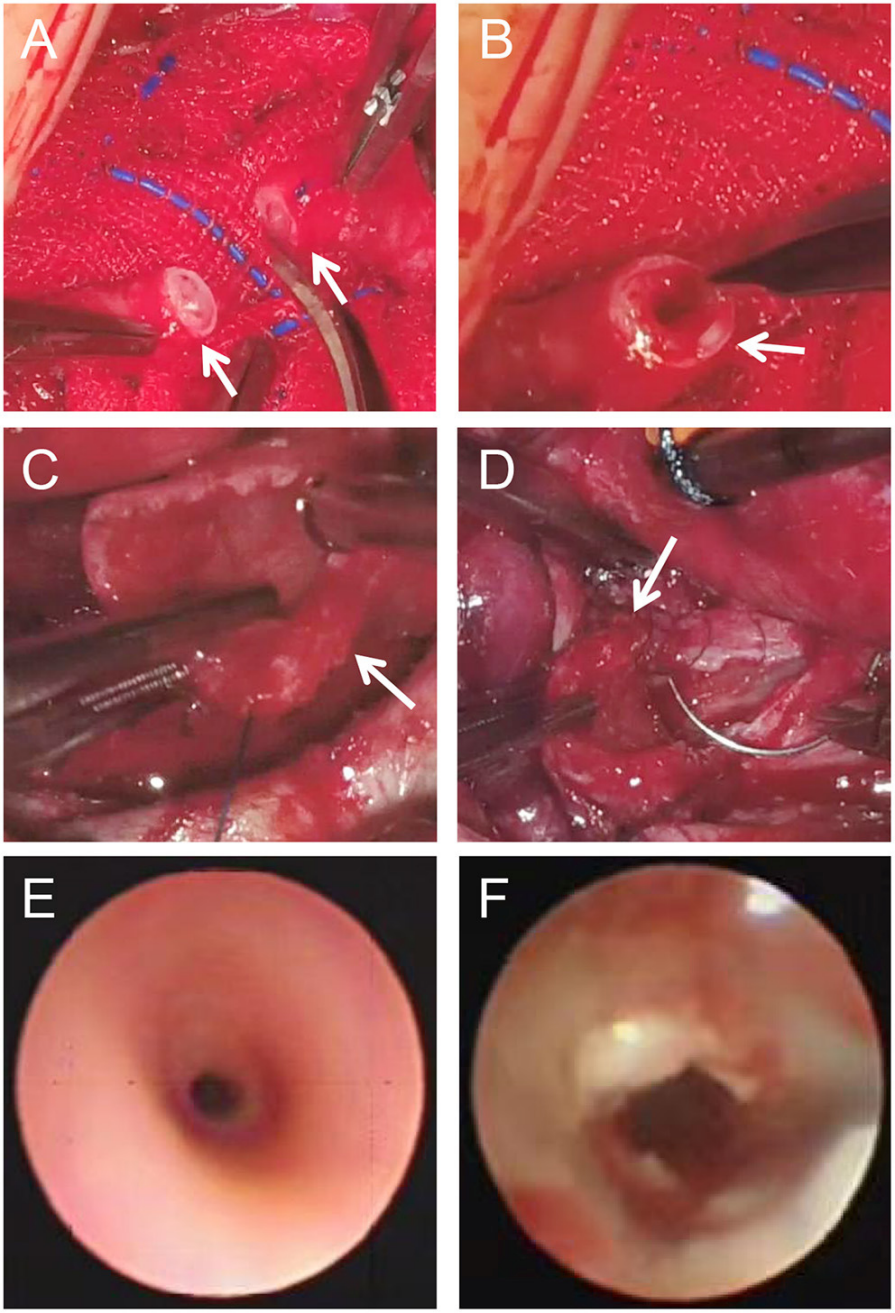
Tracheal SLIDE operation **(A–D)**: Stenotic site was surgical resected (A, arrows), the upper (B, arrow) and lower part (C, arrow) were longitudinally extended and repaired by SLIDE anastomosis (D, arrow). Representative images of surgically treated trachea [before **(E)** and after **(F)**].

**Figure 3:**
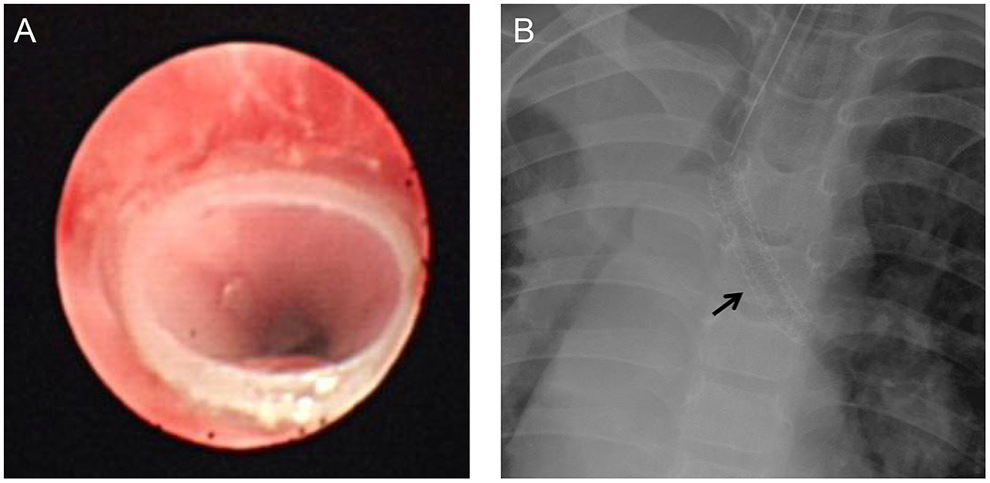
Post-operative image of fiberoptic bronchoscopy stent implantation **(A)** and chest X-ray [**(B)**, arrow].

In the airway stenosis group, most cases have no obvious clinical symptoms such as dyspnea before surgery. (i) Seven cases underwent SLIDE tracheoplasty concomitantly with primary surgery due to severe airway stenosis (Figure 2); (ii) four cases with tracheal stent implantation or bronchiectasis (Figure 3); (iii) four cases with tracheal intubation passing through the stenotic site to support softening collapse of the narrowed trachea; (iv) six cases with pure prolonged mechanical ventilation, fully discharge secretion and infection control; and (v) two cases with a tracheotomy to fully discharge secretions, control infection, and intermittently and gradually break away from the ventilator.

*Diaphragm Dysfunction Group*

**Figure 4:**
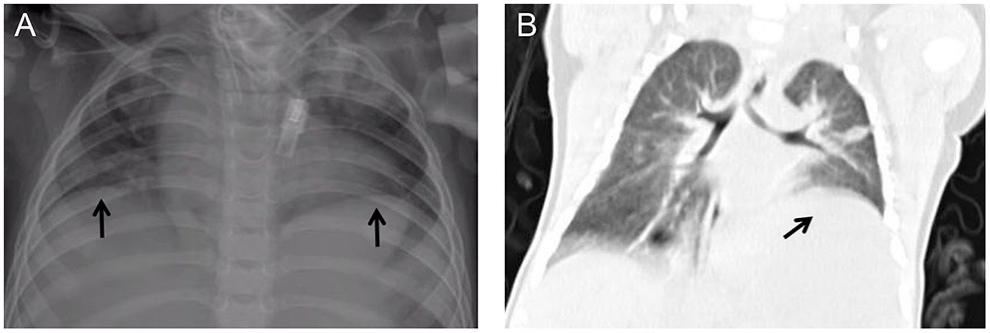
Post-operative chest X-ray **(A)** and computed tomography in coronal view **(B)** showing the unilateral diaphragmatic elevation (arrows).

In the diaphragm dysfunction group, clinical symptoms of phrenic nerve palsy after cardiac surgery varied in severity, ranging from asymptomatic conditions to life-threatening respiratory distress. Specific strategies for postoperative diaphragmatic dysfunction determined according to the specific situation: (i) Eight cases were controlled by prolonging mechanical ventilation time, using 45 degrees up semi-recumbent position, and eliminating secretions with infection control. (ii) Five cases were treated with the above method after tracheotomy, which was suitable for bilateral diaphragmatic paralysis and in patients with severe abnormal breathing. (iii) Four cases of diaphragm fold surgery, primarily for patients with unilateral septal muscle paralysis (Figure 4), significant elevation, with apparent abnormal breathing.

*Severe Pulmonary Infection Group*

**Figure 5:**
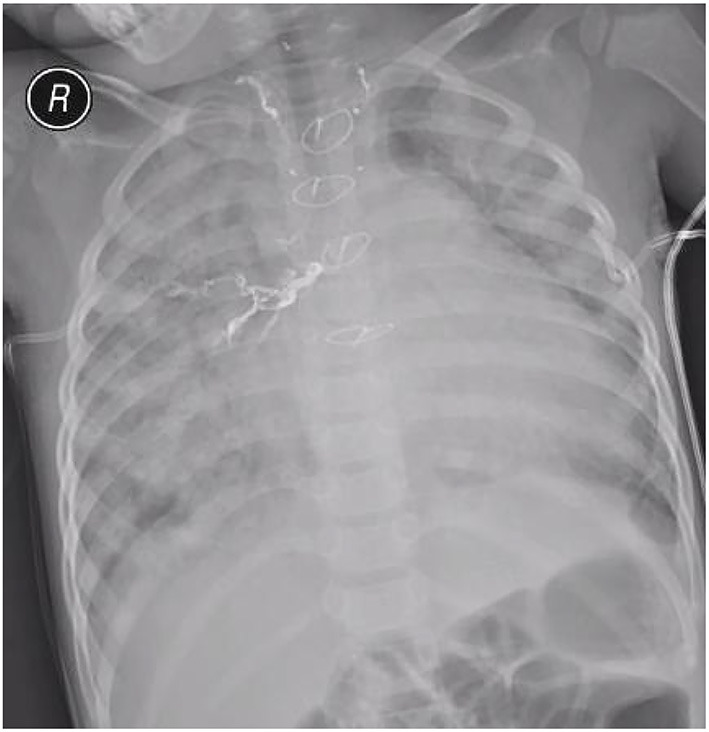
Post-operative chest X-ray showing a representative image of severe pulmonary infection.

In the pulmonary infection group (Figure 5), all cases were combined treated with postural drainage, prone ventilation, and full sputum discharge. According to the etiological examination, antibiotics were reasonably used to control infection. Combined with step-down weaning by raising the upper body position, high-frequency breathing apparatus was provided based on the clinical conditions following tracheal intubation removal, where the patients were all successfully weaned off.

### Mechanical Ventilation Duration

The patients all recovered and were discharged from the hospital. The duration of wearing a ventilator ranged from 8 to 55 days (18.6 ± 12.1). Notably, diaphragmatic dysfunction had the longest time and was more challenging during weaning (33.6 ± 13.9 days). Even though severe lung infection is the most prevalent cause for difficult weaning, it was associated with shorter ventilator use (11.9 ± 3.8 days), while the intubation time in the airway stenosis group was 17.7 ± 9.0 days.

## Discussion

In this multicenter retrospective investigation, we examined the clinical characteristics and outcomes of congenital heart diseases patients with weaning difficulties, then identified the leading causes associated with this procedure and summarized individualized treatment experiences.

Weaning challenges in pediatric patients (neonates and toddlers) with congenital heart disease seriously affects recovery after surgery. After excluding the weaning failure caused by cardiac surgery, we found out the most critical reasons affecting the weaning failure in pediatric patients with congenital heart diseases, although we were unable to establish direct causality between any of these variables and the weaning procedure. Similar findings were previously identified in patients with diverse heart lesions (11, 12).

**Figure 6:**
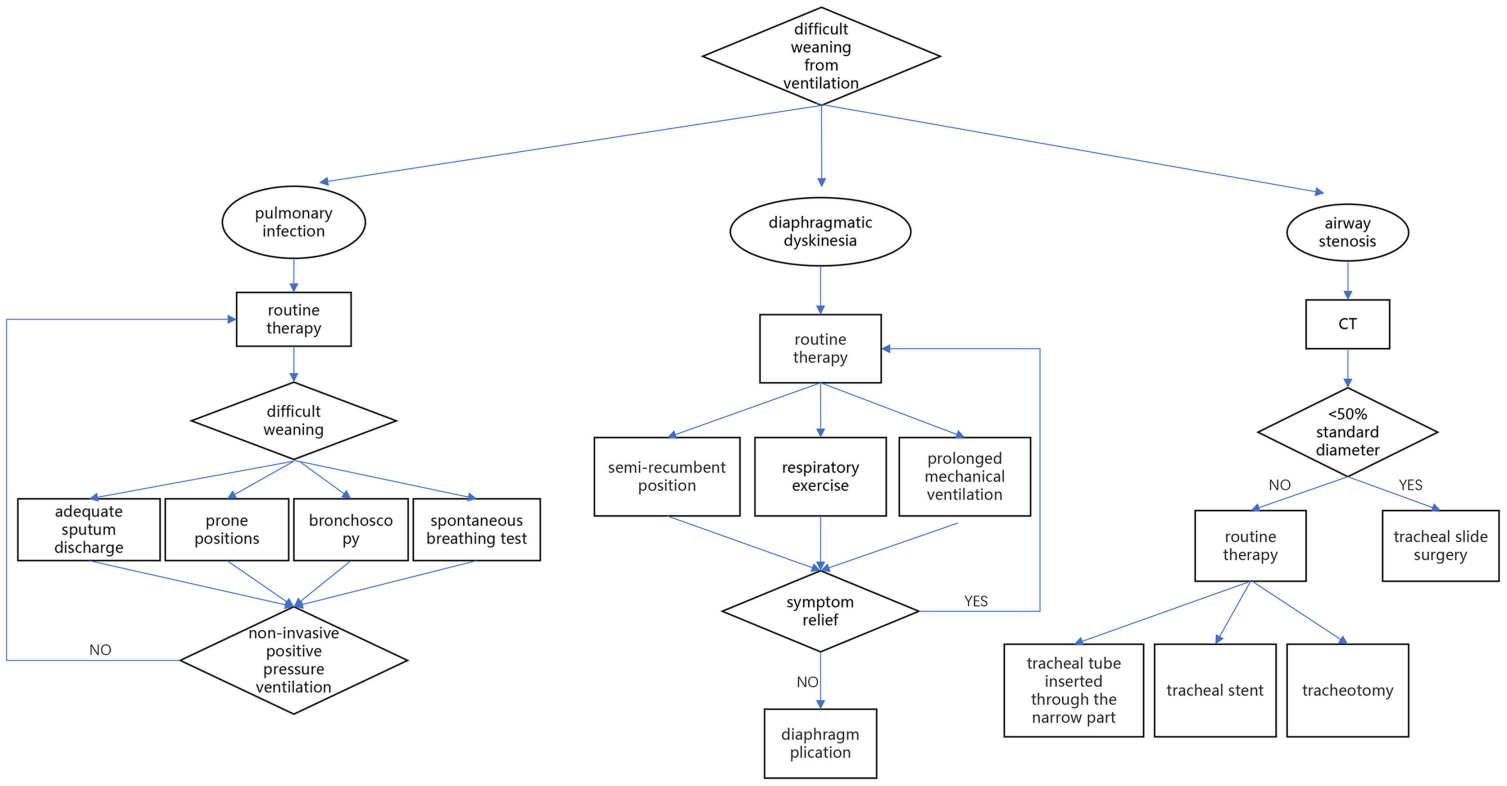
The flow chart of the three strategies adopted in the three situations.

The novelty of our investigation is its specific focus on the individualized treatment strategies. An effective strategy to minimize weaning difficulty should include identification who has or may has a difficult weaning, preemptive recognition of situations at increased risk of weaning failure and appropriate individualized treatment planning. A flow chart of the three strategies adopted in the three situations was summarized in Figure 6. The following is a summary of individualized treatment experiences for three conditions:

*Airway Stenosis*

Airway stenosis can be divided into congenital and secondary stenosis. Congenital stenosis can exist alone and is accompanied by cardiac vascular malformation, such as double aortic arch, pulmonary artery sling, while secondary airway stenosis primarily has neoplastic obstruction and tracheobronchial compression. Approximately 0.15% of children with precordial disease have a combination of airway stenosis (16). The abnormal vascular structure and enlarged heart chamber in pediatric patients with congenital heart disease can easily mount pressure on the trachea or bronchus, causing tracheomalacia (17). The incidence of congenital heart disease combined with tracheomalacia is 4–12%. Stenosis near the glottis can be found during endotracheal intubation, while stenosis near the carina of the lower trachea is not easily found, and CT or bronchoscopy is often required to make such diagnosis (18). Although the pressure on the trachea is usually relieved after surgery and tracheal stenosis is relieved to some extent, it is not completely relieved in a short time. Narrow airway increases breathing in children, invariably increasing cardiac load, which is particularly significant in children whose cardiac function is yet to recover after surgery.

Narrow airways hinder the discharge of secretions, and increased secretions will increase airway resistance and increase the risk of lung infection. Therefore, trachea development should be carefully evaluated before surgery, and individualized measures, timely taken. However, the clinical manifestations of airway stenosis associated with congenital heart disease are easily masked by the symptoms of the primary heart disease, and preoperative confirmation of the diagnosis relies heavily on CT and bronchoscopy Very few cases were confirmed preoperatively, but most cases were not noticed until they could not be taken offline or repeated endotracheal intubation. In this study, 20 of the 23 cases in this group were found to have different degrees of tracheal or bronchial stenosis on preoperative CT examination. Presently, we preoperationally anticipate that patients may have airway stenoses, such as pulmonary sling, vascular ring, and enlargement of the left atrium. Usually, pulmonary CTA and three-dimensional reconstruction of the airway are performed to evaluate the degree of airway stenosis (Figure 1). Tracheal SLIDE anastomosis technique was performed during surgery (Figure 2), given preoperative CT evaluation (Figure 1) showed that the diameter of the stenotic site was <50% standard diameter for age. In this study, 7 children who had difficulty in weaning after cardiac surgery with severe tracheal stenosis were extubated within 3 weeks after tracheal slide surgery and recovered well. Given the preoperative CT results, stenotic tracheal sites ≥50% standard diameter for age can be treated selectively per patient and conditions by: (1) Prolonging the ventilation time; and (2) Tracheotomy techniques for airway management and step-down weaning exercises if the patient is on a ventilator for over 3 weeks. Significantly, in the absence of inherent tracheal stenosis, relative stenosis (trachea softening or collapse) is thus caused by enlarged blood vessels or cardiomegaly. However, if the malformation is not severe, a tracheal tube can be inserted through the narrow part to support the softened trachea for ~2 weeks. On the other hand, some cases can be treated by prolonging ventilator-assisted time; however, it might increase the risk of ventilator-associated pneumonia. If trachea collapse occurs following severe softening and is also challenging to gain recovery in a short time, a tracheal stent can be considered (Figure 3). Likewise, tracheal stents could also be considered for severe bronchomalacia collapse. If the trachea cannot be taken off the machine and a tracheal stent cannot be placed, we will consider tracheotomy after more than 3 weeks of mechanical ventilation.

*Dysmotility of Diaphragm*

Another reason for the difficulty of weaning in this study may be related to the diminished mobility of the diaphragm, and similar conclusions were reached in previous studies (19). Postoperative diaphragmatic dyskinesia is mainly due to phrenic nerve injury in the cardiac surgery (20), and postoperative chest radiographs often show one or both diaphragmatic elevations, with clinical manifestations of dyspnea and even paradoxical breathing. In children with low age, low body mass, and complex congenital heart disease, the possibility of diaphragmatic palsy should be considered first, and the diaphragm motor function should be evaluated by ultrasound and chest X-ray to clarify the diagnosis as soon as possible (21). In this study, the preoperative chest X-ray did not show diaphragm elevation in this group of children, and the postoperative chest X-ray showed unilateral diaphragm elevation in 6 cases and bilateral diaphragm elevation in 11 cases. We have found in clinical practice that the degree of bilateral diaphragmatic elevation is less than the degree of unilateral diaphragmatic elevation.

The measures we took for these children were: for patients with unilateral diaphragmatic elevation, we would first use conservative treatment with semi-recumbent position (22), postural drainage, simple prolonged mechanical ventilation, and adequate drainage of secretions, and in severe cases, we would also consider unilateral diaphragmatic folding surgery. For patients with bilateral diaphragm elevation, we will use semi-recumbent position, promote the diaphragm downward, strengthen respiratory exercise, and give some time to observe the possibility of self-recovery, if it obviously affects the respiratory status and is difficult to get off the ventilator, one side of the diaphragm will be folded first, and then exercise will be performed to gradually get off the machine, if after one side of the diaphragm is folded, the effect of getting off the machine is poor and the respiratory status does not improve significantly, then If one side of the diaphragm is folded and the breathing status does not improve significantly, the other side of the diaphragm is folded again. Among them, for those who have severe abdominal breathing, long recovery time and need longer mechanical ventilation (usually will exceed 30 days), we will consider performing tracheotomy around 3 weeks to facilitate sputum removal and airway management.

*Severe Pulmonary Infection*

The present study also found that severe preoperative pulmonary infection was one of the main causes of weaning difficulties in children after cardiac surgery, which is consistent with previous findings (12). A single-center, 2-year retrospective cohort study of critically ill pediatric patients by ChoiA et al. (23) showed that severe pulmonary infections were responsible for 74.1% of the disease factors influencing the occurrence of weaning difficulties in children, and a large cross-sectional survey of 40 comprehensive ICUs by Louise Rose et al. (24) found that severe pneumonia, acute lung injury, and severe systemic infections with sepsis were responsible for ~64% of the weaning difficulties. Children with incomplete treatment of preoperative pulmonary infection and increased postoperative pulmonary secretions are prone to ventilator-associated pneumonia, resulting in respiratory failure, difficulty in withdrawal, and prolonged mechanical ventilation. Concerning treatment with the routine use of sensitive antibiotics based on etiology and drug sensitivity tests, and regular oral care, we also emphasize using different postural drainage methods per the patients' conditions. In addition, we encourage adequate sputum discharge and suction of oral secretions, and intermittent prone positions during mechanical ventilation while using cuffed tracheal intubation (25), strict hand hygiene, disinfecting procedures, and tracheal intubation with a capsule to avoid secretions from the mouth and nose into the airway. Previous studies have shown that early fiberoptic bronchoscopy and bronchoalveolar lavage can effectively relieve clinical manifestations, pulmonary atelectasis, and airway obstruction (26). In the present study, 19 children had relief of symptoms after fiberoptic bronchoscopy due to severe pulmonary arthritis (27). In the absence of contraindications, the possibility of extubation was assessed with a daily spontaneous breathing test. Non-invasive positive pressure ventilation was selected per the patient's condition after extubation, which was helpful for early weaning. In the absence of contraindications, a daily spontaneous breathing test assesses the possibility of extubation. After extubation, non-invasive positive pressure ventilation is selected based on the patient's clinical condition, which is helpful for early weaning.

### Limitations

Consideration of these findings should include acknowledgment of the limitations of our study design. First, this is a retrospective and non-randomized study, prospective intervention studies may be considered in the future. Second, there are some other factors that may affect weaning outcome and need to be verified in follow-up studies. Finally, as neonatal ventilation issues are often very different due to the congenital nature to infants, these results should be interpreted with caution.

## Conclusions

In summary, airway stenosis, diaphragm dysfunction, and severe pulmonary infection are leading causes of postoperative meaning difficulties in pediatric patients with congenital heart diseases. These factors should be taken into consideration and individualized treatment decisions should be made when providing care for such patients.

## Data Availability Statement

The original contributions presented in the study are included in the article/supplementary material, further inquiries can be directed to the corresponding author/s.

## Ethics Statement

The studies involving human participants were reviewed and approved by Ethics Committee of the Second Xiangya Hospital of Central South University. Written informed consent to participate in this study was provided by the participants' legal guardian/next of kin.

## Author Contributions

XiaW and CF drafted the manuscript. CF and JY designed the study. JC, JL, CI, XX, PH, and WZ revised the manuscript. XiaW, MW, KX, XunW, and WC were responsible for the collection of data or analysis. All authors read and approved the final manuscript.

## Funding

This work was supported by the Key project of science and technology of Hunan Province (No. 2020SK53420 to JY).

## Conflict of Interest

The authors declare that the research was conducted in the absence of any commercial or financial relationships that could be construed as a potential conflict of interest.

## Publisher's Note

All claims expressed in this article are solely those of the authors and do not necessarily represent those of their affiliated organizations, or those of the publisher, the editors and the reviewers. Any product that may be evaluated in this article, or claim that may be made by its manufacturer, is not guaranteed or endorsed by the publisher.
